# Discovery of Antibacterial Dietary Spices That Target Antibiotic-Resistant Bacteria

**DOI:** 10.3390/microorganisms7060157

**Published:** 2019-05-29

**Authors:** Dan Zhang, Ren-You Gan, Arakkaveettil Kabeer Farha, Gowoon Kim, Qiong-Qiong Yang, Xian-Ming Shi, Chun-Lei Shi, Qi-Xia Luo, Xue-Bin Xu, Hua-Bin Li, Harold Corke

**Affiliations:** 1 Department of Food Science & Technology, School of Agriculture and Biology, Shanghai Jiao Tong University, Shanghai 200240, China; zhang.dan@sjtu.edu.cn (D.Z.); farhatintu@sjtu.edu.cn (A.K.F.); gowoon_kim@sjtu.edu.cn (G.K.); yangqiongqiong@sjtu.edu.cn (Q.-Q.Y.); xmshi@sjtu.edu.cn (X.-M.S.); clshi@sjtu.edu.cn (C.-L.S.); 2 State Key Laboratory for Diagnosis and Treatment of Infectious Diseases, Collaborative Innovation Center for Diagnosis and Treatment of Infectious Diseases, Zhejiang University, Hangzhou 310003, China; qixia_luo@zju.edu.cn; 3 Department of Microbiology, Shanghai Municipal Center for Disease Control and Prevention, Shanghai 200336, China; xxb72@sina.com; 4 Guangdong Provincial Key Laboratory of Food, Nutrition and Health, Guangdong Engineering Technology Research Center of Nutrition Translation, Department of Nutrition, School of Public Health, Sun Yat-Sen University, Guangzhou 510080, China; lihuabin@mail.sysu.edu.cn

**Keywords:** spice extracts, drug resistant bacteria, antibacterial activity, antioxidant activity, total phenolic content, correlation

## Abstract

Although spice extracts are well known to exhibit antibacterial properties, there is lack of a comprehensive evaluation of the antibacterial effect of spices against antibiotic-resistant bacteria. In the present study, ethanolic extracts from a total of 67 spices were comprehensively investigated for their in vitro antibacterial activities by agar well diffusion against two common food-borne bacteria, *Staphylococcus aureus* and *Salmonella enteritidis*, with multi-drug resistance. Results showed that *S. aureus* was generally more sensitive to spice extracts than *S. enteritidis*. Of the 67 spice extracts, 38 exhibited antibacterial activity against drug-resistant *S. aureus*, while only four samples were effective on drug-resistant *S. enteritidis*. In addition, 11 spice extracts with inhibition zones greater than 15 mm were further verified for their broad-spectrum antibacterial properties using another 10 drug-resistant *S. aureus* strains. It was found that five spice extracts, including galangal, fructus galangae, cinnamon, yellow mustard seed, and rosemary, exhibited the highest antibacterial capacity. Further cytotoxicity of these 11 spices was determined and LC_50_ values were found to be more than 100 μg/mL except for galangal, rosemary, and sage, whose LC_50_ values were 9.32 ± 0.83, 19.77 ± 2.17, and 50.54 ± 2.57, respectively. Moreover, the antioxidant activities (ferric-reducing antioxidant power (FRAP) and trolox equivalent antioxidant capacity (TEAC) values) and total phenolic content (TPC) of spice extracts were determined to establish possible correlations with the antibacterial activity. Although the antibacterial effect was positively correlated with the antioxidant activities and TPC, the correlation was weak (*r* < 0.5), indicating that the antibacterial activity could also be attributed to other components besides antioxidant polyphenols in the tested spice extracts. In conclusion, dietary spices are good natural sources of antibacterial agents to fight against antibiotic-resistant bacteria, with potential applications as natural food preservatives and natural alternatives to antibiotics in animal feeding.

## 1. Introduction

Food poisoning caused by food-borne bacteria is one of the critical threats to human health all over the world [1]. The emergence of multi-drug resistant bacteria induced by the abuse of antibiotics cause greater obstacles for the treatment of food-borne diseases [2]. Antibiotic-resistant bacteria, such as *Staphylococcus* (*S.*) *aureus* and *Salmonella* (*S.*) *enteritidis*, have frequently been reported to cause contamination of different foods like raw pork, beef, and poultry [3,4]. Many attempts, such as the use of synthetic preservatives, have been used to control microbial growth and ensure food safety. However, there have potential carcinogenic and toxicological properties, as well as side effects like food allergies and sensitivities that are harmful to human health [5]. To counter these problems, much effort has gone into the search for “naturally derived” alternative antimicrobials since plants are known to produce diverse secondary metabolites that are associated with anti-infective mechanisms against the invasion of pathogenic microorganisms [6,7]. Among them, plant-derived spices and extracts containing a mixture of active ingredients have received growing attention, not only for their effective antibacterial activity but also for the relative difficulty in developing resistance to them. Moreover, spice and their major components are generally recognized as safe (GRAS) with no historical records of detrimental impacts and with modern toxicological verification [8].

Although spices have been widely used in rituals, and as flavorings and coloring agents since ancient times [9], recent literature has increasingly reported on the antibacterial activity of spices against common Gram-positive and Gram-negative bacteria responsible for human infectious diseases and food safety problems [10,11,12,13]. Examples of such spices are cinnamon, oregano, nutmeg, basil, pepper, thyme, clove, rosemary, ginger, cumin, etc. However, few studies have focused on the inhibitory effects of these spices on antibiotic-resistant bacteria. The methanolic and ethanolic extracts of cinnamon, which was the most studied spice, were reported to have inhibitory effects on high level gentamicin-resistant (HLGR) enterococci, multi-drug resistant *Escherichia coli* AG100, methicillin-resistant *S. aureus* (MRSA), as well as β-lactamase producing multi-drug *Klebsiella pneumonia* and *Pseudomonas aeruginosa* [14,15,16]. Moreover, the antibacterial properties of spices are mostly attributed to lipophilic essential oils in most previous studies [17]. However, spices are also rich in hydrophilic antioxidants [18], such as polyphenols, many of which possess excellent antioxidant activity, and also exhibit good antibacterial activity [19]. Considering that microbial contamination and lipid oxidation are the two major factors resulting in food spoilage [20], spice hydrophilic extracts with good antibacterial and antioxidant activities can be promising natural food preservatives. For instance, extracts of cinnamon, oregano, and especially clove, were confirmed to be effective for retarding lipid oxidation and reducing pathogen numbers in real food matrices like cheese and raw pork [21,22]. More importantly, probiotic bacteria like lactic acid bacteria (LAB) were less influenced by the presence of these phenolic rich spice extracts, indicating that spice extracts could be applied in foods not only to prolong shelf-life but also enhance health benefits of foods [23].

Therefore, the aim of this study was to evaluate systemically and compare the in vitro antibacterial activity of the ethanolic extracts of 67 spices, mainly focusing on their effects on antibiotic-resistant bacteria, and to analyze the correlation among antibacterial activity, antioxidant activity, and total phenolic content (TPC) in spices. Overall, this study can shed light on the control of antibiotic-resistant bacteria using dietary spices, which should have broad applications in food industry to help assure food safety.

## 2. Materials and Methods

### 2.1. Chemicals and Reagents

2,2-azinobis (3-ethylbenzothiazoline-6-sulfonic acid) diammonium salt (ABTS) and 2,4,6-tri(2-pyridyl)-s-triazine (TPTZ) was from Sigma/Aldrich (St. Louis, MO, USA). Gallic acid was from Energy Chemical (Shanghai, China). Dimethyl sulfoxide (DMSO) was from Beyotime (Shanghai, China), Folin–Ciocalteu reagent was from Macklin (Shanghai, China), 6-hydroxy-2,5,7,8-tetramethylchromane-2-carboxylic acid (trolox) was from Fluka Chemika AG (Buchs, Switzerland). Hydrochloric acid, iron (III) chloride hexahydrate and Iron (II) sulfate heptahydrate were from Sinopharm Chemical Reagent (Shanghai, China), acetic acid and sodium acetate were from Molbase (Shanghai, China). Potassium persulphate and ethanol were obtained from Titanchem (Shanghai, China). Sodium carbonate and methanol were purchased from J&K (Beijing, China). Luria Bertani (LB) broth, agar bacteriological, and Mueller–Hinton (MH) broth were purchased from Oxiod (Basingstoke, England). Antibiotics, including ampicillin, cefazolin, ciprofloxacin, clindamycin, erythromycin, gentamicin, oxacillin, penicillin, streptomycin, sulfisoxazole, and tetracycline were purchased from Meilune (Dalian, China). All chemicals used in the experiment were of analytical grade.

### 2.2. Spice Materials

The 67 dried edible spice materials were purchased from the local markets in Shanghai, China. The basic information (scientific name and common name) of these spices is detailed in Table 1.

### 2.3. Microorganisms and Culture

Two strains of *Salmonella enteritidis* (drug-resistant *S. enteritidis* SJTUF 10987 and the standard strain *S. enteritidis* ATCC 13076) and 12 strains of *Staphylococcus aureus*, including 11 drug-resistant strains *S. aureus* SJTUF 20745, *S. aureus* SJTUF 20746, *S. aureus* SJTUF 20755, *S. aureus* SJTUF 20758, *S. aureus* SJTUF 20772, *S. aureus* SJTUF 20827, *S. aureus* SJTUF 20841, *S. aureus* SJTUF 20862, *S. aureus* SJTUF 20973, *S. aureus* SJTUF 20978, and *S. aureus* SJTUF 20991, as well as the standard strain *S. aureus* ATCC 25923 were used in this study. These strains were stored at −80 °C. To prepare the inocula, a single colony of the bacteria grown on the LB agar plate was selected and transferred into the LB broth to culture overnight in a rotary incubator (37 °C, 150 rpm). The bacterial suspension was then diluted to approximately 1 × 10^6^ colony-forming units (CFU)/mL for subsequent antibacterial experiments. 

### 2.4. Verification of the Drug-Resistant Bacteria

The verification assay was undertaken using the agar dilution method following the testing procedures of the Clinical and Laboratory Standards Institute, USA (CLSI M100-S28, 2018). Briefly, tested strains were cultured in MH broth overnight, then centrifuged (1000× *g*, 1 min) and transferred to a custom 96-well microtitre plate using 1 mL of 0.85% sodium chloride solution. The absorbance of bacterial resuspension at OD 600 was adjusted to 0.5, followed by further 100 fold dilution to make a final concentration of 1 × 10^6^ CFU/mL. Subsequently, bacterial suspensions were incubated to the MH agar plates which were supplemented with antibiotics under prescribed breakpoint concentrations stated by CLSI using the multipoint incubator (HM1-12-001, Hen Gao Technology Development Co., Ltd., Tianjin, China), and the plates were cultured at 37 °C for 20 h. In addition, the oxacillin agar dilution method was used for the detection of MRSA (CLSI, 2018). Isolates were considered to be multi-drug resistant if they were resistant to at least three different categories of antibiotics, based on their growth condition on the MH plates.

### 2.5. Preparation of Spice Ethanolic Extracts

The clean dietary spices were air-dried in a ventilated oven at 40 °C for 24 h, then ground into fine powders by a miller (S025, IKA, Staufen, Germany). Powdered samples (4.0 g) were extracted with 80 mL of 80% (*v*/*v*) ethanol in a shaking bath (MQT-50, Shanghai Min Quan Co., Ltd., Shanghai, China) at room temperature (23 ± 1 °C) for 24 h. In this study, 80% ethanol was used for the extraction, since ethanol is of relative low toxicity among several organic solvents, and 80% ethanol was efficient to extract antioxidant and antibacterial components from spices and herbs based on our previous study [22]. Afterwards, the mixture was centrifuged at room temperature (900× *g*, 15 min) and the collected supernatant was concentrated by a rotatory vacuum evaporator (RE-52AA, Shanghai Ya Rong Co., Ltd., Shanghai, China) at 40 °C, then the concentrated extract was dried by a vacuum freeze-dryer (SJIA-5FE, Ningbo Shuang Jia instrument Co., Ltd., Ningbo, China). The freeze-dried samples were stored at −20 °C in small vials for further use.

### 2.6. Determination of Antibacterial Activity

The inhibitory effects of 67 spices were estimated according to the agar diffusion method as previously reported with slight modification [24]. The freeze-dried extracts were dissolved in DMSO to a final concentration of 100 mg/mL and filtered through 0.22 μm sterilizing filters. Briefly, all bacteria were diluted to about 1 × 10^6^ CFU/mL with sterile LB medium, and then 100 µL of each bacterial suspension was evenly spread onto the surface of LB agar plate by sterile glass beads (6 mm in diameter). Oxford cups (sterilized hollow cylinder with an inner diameter of 6 mm, outer diameter of 7.8 mm, and height of 10 mm) were placed lightly on the agar surface, and then 60 µL of the prepared samples (100 mg/mL) were delivered into the cups. DMSO (60 µL/cup) was used as a negative control. The plates were incubated at 37 °C for 24 h for bacterial growth in an incubator (BI-150A, Shanghai Stik Co., Ltd., Shanghai, China). The diameters of the inhibitory zones (DIZs) formed around the Oxford cups were measured to evaluate the antibacterial activity and expressed in millimeter (mm). All experiments were performed in triplicate. DIZ values less than 8 mm were considered as “no inhibition zone (NIZ)”.

### 2.7. Minimum Inhibitory Concentration (MIC) and Minimum Bactericide Concentration (MBC) Assays

Minimum inhibitory concentration (MIC) and minimum bactericide concentration (MBC) were determined according to the method described by Elshikh et al. with minor adjustment [25]. Briefly, 100 μL of MH broths were added into 96-well plate and then another 100 μL of dissolved samples, whose initial concentrations were 100 mg/mL, were added in first wells. Serial two-fold dilutions were made with final concentrations ranging from 50 mg/mL to 0.39 mg/mL. Afterwards, 100 μL of the standardized bacteria suspensions (1 × 10^6^ CFU/mL) was added to each test well, so that the final volume in each well was 200 μL. Plates were incubated at 37 °C for 24 h. After incubation, 30 μL of the freshly prepared resazurin (0.015%) was added to all test wells, and further incubated for 2 h to allow the viable microorganism to metabolize the blue resazurin dye into pink resorufin. MIC was defined as the concentration at which the corresponding well showed no color change. Afterwards, the contents of wells with concentrations equal or higher than MIC values were directly incubated onto the MH plate, and the lowest concentration at which there was no colony growth was defined as MBC.

### 2.8. Cytotoxicity of Spice Extracts

Human foreskin fibroblast (HFF) cells were grown in DMEM medium supplemented with 10% fetal bovine serum (FBS), and maintained at 37 °C in a humidified 5% CO_2_ incubator. The toxicity of spice extracts was evaluated by 3-(4,5)-dimethylthiazol-2-yl)-2,5-diphenyltetrazolium bromide (MTT) assay as described by Mosmann with slight modification [26]. HFF cells were seeded at a density of 5 × 10^4^ cells/mL into the 96-well plate and incubated overnight to adhere the cells. The cells were treated with various concentrations (5–100 µg/mL, and the concentration of DMSO was diluted below 0.1%) of different extracts and incubated at 37 °C in a humidified 5% CO_2_ incubator for 24 h. The untreated cells were included as control. After the incubation period, MTT (20 µL of 5 mg/mL) was added into each well and incubated for 4 h. The formazan crystals were dissolved with DMSO (100 µL). The absorbance was measured at 570 nm using a microtitre plate reader (SpectraMax iD3, Molecular Devices, Silicon Valley, NC, USA). The LC_50_ value was calculated as the concentration of the extract that resulted in 50% reduction of absorbance compared to control cells [27].

### 2.9. Determination of Antioxidant Capacity

#### 2.9.1. Ferric-Reducing Antioxidant Power (FRAP) Assay

The ferric-reducing antioxidant power (FRAP) assay was carried out according to the procedures described by Gan et al. [28]. Briefly, the FRAP working solution was freshly prepared before the experiment, with sodium acetate solution (300 mM, pH = 3.7), TPTZ (10 mM solved with HCl) and ferric chloride solution (20 mM) mixedin a volume ratio of 10:1:1, respectively. The FRAP working solution was then incubated at 37 °C before use. The proper dilutions (100 µL) of samples were added to 3 mL of the FRAP working solution and their absorbance at 593 nm was determined after incubation for 4 min at room temperature (23 ± 1 °C). Ferrous sulfate solution (0.1–1 mM) was used as the standard for the calibration curve, and the results were expressed as mmol Fe(II)/g dry weigh (DW) extract powder. All tests were performed in triplicate.

#### 2.9.2. Trolox Equivalent Antioxidant Capacity (TEAC) Assay

The trolox equivalent antioxidant capacity (TEAC) assay was carried out to determine the free radical scavenging capacity using ABTS^+^ according to the method previously reported [28]. ABTS stock solution was prepared by mixing 7 mM ABTS and 2.45 mM potassium persulfate in a ratio of 1:1 (*v*/*v*) and then incubated at room temperature (23 ± 1 °C) in the dark for at least 16 h. The ABTS working solution was prepared by dilution of the stock solution with 80% ethanol before use, and then the absorbance at a wavelength of 734 nm was adjusted to 0.7 ± 0.05. The blank control is a mixture of 3.9 mL ABTS working solution and 0.1 mL of 80% ethanol. The spice extract sample (0.1 mL) was diluted with 80% ethanol to provide 20–80% inhibition of the blank absorbance, and then the properly diluted samples were added to 3.9 mL ABTS working solutions and mixed thoroughly. The absorbance of reactive mixture was determined at 734 nm after incubation at room temperature (23 ± 1 °C) for 6 min. Quantitative results were determined from the standard curve of trolox (0.05–0.8 mM) and were expressed as mmol trolox/g dry weight (DW) extract powder. All tests were performed in triplicate.

### 2.10. Determination of Total Phenolic Content (TPC)

TPC was determined by the Folin-Ciocalteu method reported previously with some modification [29]. The appropriate dilutions of samples (200 µL) were mixed with 1 mL of 0.5 M Folin–Ciocalteu reagent at room temperature (23 ± 1 °C) for 4 min, and then reacted with 800 µL of saturated sodium carbonate solution (75 g/L) in dark for 2 h. Finally, the absorbance of the reaction mixtures was measured at 760 nm with a spectrophotometer (181712007PC, Shanghai Jing Hua Co. Ltd., Shanghai, China) and quantified on the base of the standard curve of gallic acid (0.01–0.1 mM). The results were expressed as milligram gallic acid equivalent (mg GAE)/g DW extract powder. All tests were performed in triplicate.

### 2.11. Statistical Analysis

All the measurements were performed in triplicate, and the results were expressed as mean ± standard deviation (SD). Statistical analysis was performed using Microsoft Excel 2016 (Microsoft, Seattle, MA, USA) and SPSS 22.0 (IBM SPSS Statistics, IBM Corp, Somers, NY, USA). Pearson linear correlation analysis and principal component analysis (PCA) were performed to analyze relationships among parameters, and *p* value less than 0.01 was defined as statistical significance.

## 3. Results

### 3.1. Verification of Drug-Resistant Bacteria

The antibiotic-resistant strains of *S. aureus* and *S. enteritidis* were isolated from food samples in our lab previously. In order to confirm their antibiotic resistance, we first tested their resistance to 11 common antibiotics from different categories. The breakpoint concentration of each antibiotic defined by CLSI and corresponding bacterial resistance spectra are shown in Table 2. All tested bacteria were resistant to antibiotics. Except for *S. aureus* SJTUF 20827 and *S. aureus* SJTUF 20973, the remaining bacteria were identified as multi-drug resistant bacteria, resistant to at least three antibiotics. These bacteria showed the highest resistance rate to erythromycin, reaching up to 83.3%, followed by ciprofloxacin (75%), Clindamycin (75%), gentamicin (50%), and streptomycin (50%). However, no strain resistant to oxacillin (methicillin-resistant *Staphylococcus aureus*, MRSA) was detected. Overall, it was confirmed that all selected bacteria were antibiotic-resistant, most of which were multi-drug resistant.

### 3.2. Antibacterial Activity against Antibiotic-Resistant Bacteria

The agar diffusion method was used to evaluate systematically the antibacterial activity of 67 spice extracts on antibiotic-resistant *S. aureus* SJTUF 20978 and *S. enteritidis* SJTUF 10987, with each standard strain used as the comparison. The results of DIZ were presented in Table 1. A significant variation in the antibacterial activity reflected by different DIZ values was observed, depending on the type of spice extracts and the subject bacteria.

For antibiotic-resistant *S. aureus* SJTUF 20978, a significant proportion of the spices (38, approximately accounting for 56.7% of the total tested samples) exhibited antibacterial activity, with DIZ in the range of 8.7–25.6 mm. Besides, 11 of these spice extracts (accounting for 16.4%) showed a relatively superior antibacterial activity, with DIZ values greater than 15 mm. Among there, galangal, the rhizome of *Alpinia galanga* (L.) Willd., showed exceptional antibacterial capacity, with the DIZ reaching 25.6 mm, followed by cinnamon, fructus galangae (ripe fruit from galangal), yellow mustard seed, rosemary, and marjoram, with DIZ values of 20.7, 20.2, 18.6, 18.3, and 17.2 mm, respectively. Moreover, the MIC and MBC results of these 11 samples were determined and were shown in Table 3. The MIC values ranged from 0.40–6.25 mg/mL, and MBC values ranged from 0.40–12.5 mg/mL, which were one or two times higher than MICs. Among them, clove fruit, sage, rosemary, and liquorice had the lowest MIC value (0.40 mg/mL), and fructus galangae, galangal, and yellow mustard seed showed a relatively high value (6.25 mg/mL). However, for drug-resistant *S. enteritidis* SJTUF 10987, the overall antibacterial effects of tested spice extracts were relatively low, and only four samples showed inhibitory activity, including cinnamon (DIZ = 16.0 mm), male clove (DIZ = 11.0 mm), thorn amomum villosum (DIZ = 8.6 mm), and female clove (DIZ = 8.5 mm).

**Figure 1:**
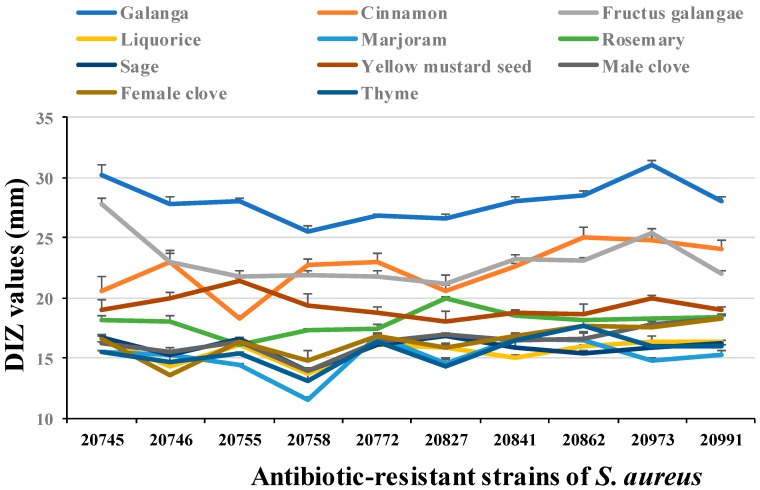
The antibacterial activity of selected spice extracts on 10 antibiotic-resistant strains of *S. aureus*.

In general, the antibacterial activity of spice extracts against antibiotic-resistant bacteria was somewhat less effective compared to corresponding standard strains *S. aureus* ATCC 25923 and *S. enteritidis* ATCC 13076 (data shown in Table 1). Besides, spice extracts showed much better antibacterial activity against Gram-positive *S. aureus* than Gram-negative *S. enteritidis*. Therefore, we further tested whether spice extracts had a broad spectrum antibacterial effect on drug-resistant *S. aureus*. 11 spice extracts with DIZ more than 15 mm on drug-resistant *S. aureus* SJTUF 20978 were selected to verify their antibacterial capacity against another 10 antibiotic-resistant strains of *S. aureus*. As shown in Figure 1, all selected spice extracts exhibited inhibitory effects against the validated antibiotic-resistant strains of *S. aureus*. Among them, galangal (DIZ = 25.6–31.1 mm), fructus galangae (DIZ = 21.2–27.8 mm), and cinnamon (DIZ = 18.3–25.1 mm) showed the strongest antibacterial effects, followed by yellow mustard seed (DIZ = 18.0–21.4 mm) and rosemary (DIZ = 16.2–19.9 mm). Overall, selected spice extracts possessed a broad spectrum antibacterial effect against antibiotic-resistant *S. aureus*.

### 3.3. Cytotoxicity of Spice Extracts

Spices were generally considered to be non-toxic or less toxic because of their natural origin and long use as food additives and medicine for ailment treatments. Meanwhile, some studies on the efficacy and safety of plants pointed out that some phytochemicals displayed certain cytotoxicity, genotoxicity, and carcinogenic effects when used chronically [30]. Therefore, it was necessary to determine the cytotoxicity of the selected 11 spice extracts with good activity against multi-drug resistant *S. aureus*. The cytotoxicity of these chosen 11 spices were determined by using the in vitro assay with HFF cells, and LC_50_ values were calculated. Of 11 spices tested, eight spice extracts did not show any cytotoxicity against HFF cells after 24 h of treatment with the highest concentration tested (100 μg/mL), suggesting that their LC_50_ values higher than 100 μg/mL. However, other three spices, including galangal, rosemary, and sage, were able to inhibit the growth of HFF cells at the LC_50_ of 9.32 ± 0.83, 19.77 ± 2.17, and 50.54 ± 2.57 μg/mL, respectively, indicating their potential safety issue. Overall, the results found that most spice extracts with high antibacterial effect were low toxic, and could be used as potential antimicrobial agents in food industry.

### 3.4. Antioxidant Activity of Spice Extracts

The antioxidant activity of spice extracts was determined using FRAP and TEAC assays, and the results are shown in Table 4. The strongest antioxidant activity determined by FRAP assay was found in the extract of female clove, followed by male clove, allspice, red pepper, and fructus amomi, showing the FRAP values about 6682 ± 68.6, 5453 ± 23.9, 4404 ± 23.9, 4137 ± 147 and 3605 ± 201 mmol Fe (II)/g DW extract powder. In addition, the antioxidant activity measured by TEAC method was also highest in the extract of female clove, followed by male clove, semen alpiniae katsumadai, allspice, and fructus amomi. The TEAC values were 3415 ± 53.1, 3131 ± 177, 2662 ± 83.7, 2184 ± 43.9, and 2153 ± 370 mmol Trolox/g DW extract powder, respectively. Besides, the lowest antioxidant activity was found in the extract of dried lemon, with FRAP and TEAC values about 50.3 mmol Fe (II)/g DW extract powder and 17.4 mmol Fe (II)/g DW extract powder, respectively.

### 3.5. TPC of Spice Extracts

The TPC in spice extracts was analyzed through the Folin-Ciocalteu method, and varied from 7.35 to 485 mg GAE/g DW extract powder (Table 4). The highest TPC was observed in the extract of female clove, followed by semen alpiniae katsumadai, male colve, red pepper, and fructus amomi, and the TPC values were 485 ± 18.5, 473 ± 8.67, 424 ± 14.9, 378 ± 3.52, and 360 ± 6.80 mg GAE/g DW extract powder, respectively. Additionally, the lowest TPC was found in the extract of dried lemon (7.35 mg GAE/g DW), consistent with the results of antioxidant activity.

### 3.6. Correlation Analysis

In order to shed light on the potential antibacterial components in spice extracts, the correlations among antibacterial activity on *S. aureus* (indicated by the DIZ values), antioxidant activity (indicated by the FRAP and TEAC values), and TPC were analyzed. As shown in Table 5, a strong positive correlation was found between TPC and FRAP/TEAC values (*r* > 0.9, *p* < 0.01), suggesting that polyphenols mainly contributed to the antioxidant activity of spice extracts. However, regarding the relationship between DIZ values and FRAP/TEAC values, a significant but weak positive correlation (*r* < 0.5, *p* < 0.01) was observed, indicating that antioxidant activity of spice extracts was only slightly related to their antibacterial capacity. Besides, DIZ values were also found to be weakly correlated with TPC (*r* = 0.541 and 0.568, *p* < 0.01), suggesting that polyphenols were only partly responsible for the antibacterial activity of spice extracts on *S. aureus*, and some other substances should exist in spice extracts to contribute to their overall antibacterial activity.

### 3.7. Principal Component Analysis

**Figure 2:**
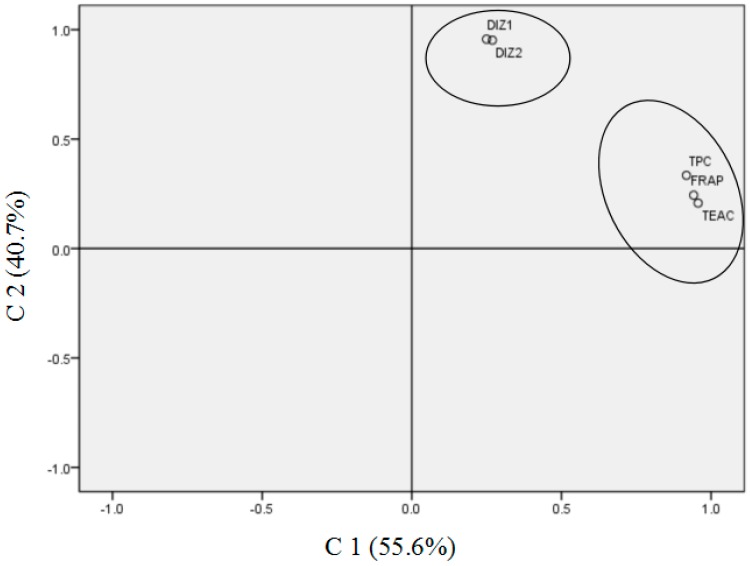
The results of principal component analysis (PCA). C1 included the factors total phenolic content (TPC), ferric-reducing antioxidant power (FRAP), and trolox equivalent antioxidant capacity (TEAC), suggesting that TPC was closely related to the antioxidant capacity. C2 contained DIZ values of *S. aureus* ATCC 25923 (DIZ1) and drug resistant *S. aureus* SJTUF 20978 (DIZ2). C1 and C2 were clearly divided into two separate clusters, suggesting that there was no evident relationship of antibacterial capacity with antioxidant ability and TPC.

To further analyze the relationships among antibacterial activity, antioxidant activity, and TPC of spice extracts and to select suitable spice extracts with good antioxidant and antibacterial activities as food preservative candidates, principal component analysis (PCA) was performed to cluster factors, including TPC, FRAP, TEAC, and DIZ values against both *S. aureus* ATCC 25923 and *S. aureus* SJTUF 20978 (Figure 2). According to the results of Kaiser–Meyer–Olkin (KMO) and Bartlett’s test (KMO value = 0.725, *p* < 0.05), as well as the communalities of factors with extraction >0.94, the data met the requirements of PCA. In addition, the cumulative variance contribution rate of the two main components (C1 and C2) extracted was 96.3%, with C1 counting for 55.6% and C2 being 40.7%. According to the rotated component matrix, C1 included the factors TPC, FRAP, and TEAC, suggesting that TPC was closely related to the antioxidant capacity. On the other hand, C2 contained DIZ values of *S. aureus* ATCC 25923 (DIZ1) and drug-resistant *S. aureus* SJTUF 20978 (DIZ2). More interestingly, C1 and C2 were clearly divided into two separate clusters (Figure 2), indicating there was no evident relationship of antibacterial capacity with antioxidant ability and TPC. These results were generally in agreement with those of the correlation analysis.

Next, the general score (GS) of each sample was calculated based on the factor scores of the two principal components, following the equation GS = (C1 × 0.556 + C2 × 0.407)/0.963, and the results are shown in Table S1. Spice extracts with higher GS usually exhibited higher phenolic contents, antioxidant, and antibacterial activity. Based on the results listed in Table S1, clove extracts, prepared from both fruit and flower of clove, as well as cinnamon showed the highest GS values, indicating that they could be used as potential promising food preservatives by means of reducing microbial contamination and lipid spoilage oxidation simultaneously.

## 4. Discussion

In this study, 38 out of 67 tested spices displayed various degrees of antibacterial activity against antibiotic-resistant strain of *S. aureus*, while only four were effective against drug-resistant strain of *S. enteritidis*. The antibacterial activity seemed to be bacteria-dependent, and Gram-positive bacteria were more susceptible to the tested spice extracts than Gram-negative bacteria, which was in accordance with many previous studies [31,32]. Different from Gram-positive bacteria, Gram-negative bacteria have an outer membrane rich in lipopolysaccharides, as well as a unique periplasmic space. The complex composition and spatial structure of lipopolysaccharides form a barrier for penetration of antimicrobial agents, besides, the presence of enzymes in periplasmic space may break down intrusive molecules, preventing the antibacterial drugs entering intracellular environment [29]. Additionally, the antibacterial activity of certain spice extracts tested in our study was also reported by previous studies. For instance, chilli, lemongrass, bay leaf, cumin, cinnamon, clove, parsley, basil, sage, thyme, rosemary, and mint, were all demonstrated to show antibacterial capacity against *S. aureus* [33,34,35,36]. However, considering the difference in extraction solvent, extraction method, and dosage of samples, it is difficult to directly compare these results with the results of our present study. More importantly, the inhibitory effects of spice extracts on multi-drug resistant bacteria were relatively less reported. Gull et al. revealed that eight drug-resistant bacteria were inhibited by ginger extract at a concentration of 100 mg/mL, with the DIZ ranging from 11 to 15 mm [37]. Mandal et al. reported that the DIZ value obtained from ethanol extracts (20 µL, 10 mg/mL) of cinnamon, clove, and cumin against methicillin-resistant *S. aureus* was in the range of 22–27 mm, 19–23 mm, and 9–15 mm, respectively [38]. Similarly, Revati et al. found that high level gentamicin-resistant enterococci isolates were sensitive to ethanol extracts (50 µL, 100 mg/mL) of cinnamon, ginger, clove, and cumin, with the DIZ values of 31–34, 27–30, 25–26, and 19–20 mm, respectively [14]. Even though, most of the previous investigations were carried out with a limited number of antibiotic-resistant bacterial isolates as well as the tested spice samples, thus the broad antibacterial spectra of spice extracts could not be demonstrated. In addition, we did not set a positive control, such as antibiotics, mainly with two reasons. On the one hand, the antibiotic resistance of bacterial strains used in our study was determined using 11 different antibiotics (Table 2). On the other hand, we were not intended to compare the antibacterial activity of these 67 spice extracts with antibiotics, since the effects of the crude extracts were generally not comparable to pure antibiotics. Besides, in our study, we used the stock concentration of extracts at a relatively high concentration, 100 mg/mL, for the DIZ evaluation, since our samples were dissolved in DMSO, which also possessed an antibacterial effect at a relatively high concentration, such as more than 5%. To rule out the interference of DMSO during subsequent MIC and MBC assays, it was necessary to increase the stock concentration of extracts to reduce the concentration of DMSO in the final working solution of samples.

In our present study, we further chose 11 spices whose DIZ values were higher than 15 mm to verify their antibacterial effects on another ten antibiotic-resistant strains of *S. aureus*, since tested spice extracts exhibited much better antibacterial activity on Gram-positive *S. aureus* than Gram-negative *S. enteritidis*, and found that galangal, fructus galangae, cinnamon, yellow mustard seed, and rosemary overall had the best antibacterial effect, and could probably be developed into antimicrobial agents. Our study may be the first large-scale investigation on the antibacterial effect of spice hydrophilic extracts on antibiotic-resistant bacteria. Therefore, this study can give a clear comparison of the antibacterial activity of spice extracts, especially against antibiotic-resistant bacteria. To provide useful information like safety for further use of these spice extracts, HFF cells were used to evaluate the cytotoxicity of them by MTT assays. All spices except galangal, rosemary, and sage were with low toxicity with LC_50_ values higher than 100 μg/mL. It was worth noting that galangal, which exhibited excellent antibacterial activity among tested spices was also found to show some cytotoxicity against HFF cells in vitro, while its toxicity should also be evaluated in in vivo studies in the future before reaching the conclusion on its toxicity. Discarding crude extracts with good antimicrobial activity only based on the in vitro cytotoxic experiments should involve caution, since cytotoxic compounds might not necessarily be the same antibacterial compounds in some cases [27]; therefore, the main antibacterial and cytotoxic compounds of galangal ethanol extracts should be further isolated and identified in the future before a final conclusion can be made.

In addition to microbial contamination, lipid oxidation is another major cause of food spoilage, therefore, we also measured the antioxidant capacity of 67 spice extracts. The antioxidant activity of tested 67 spice extracts determined by both FRAP and TEAC assays were in the range of 50.3–6682 mmol Fe(II)/g DW and 17.4–3415 mmol trolox/g DW extract powder, respectively. Among them, clove showed the highest antioxidant capacity, even comparable to butylated hydroxyanisole (BHA), an antioxidant commonly applied in food industry preservation due to its excellent hydrogen-donating capacity and metal-chelation ability [39]. Additionally, the results of PCA analysis showed that the extract of clove (both fruit and flower) and cinnamon were spotlighted as potential good candidates as natural food preservatives due to their excellent antibacterial and antioxidant properties. Several other spice extracts like coriander, cinnamon, oregano, mustard, holy basil, and green pepper were also reported to be potent food preservatives [40,41,42,43,44]. Indeed, some studies demonstrated the potential application of clove extracts in raw chicken meat and raw pork during storage to extend shelf-life, in terms of reducing microbes, maintaining natural color, and retarding lipid oxidation [40]. The antimicrobial and antioxidant activities of clove were mainly attributed to the presence of secondary metabolites. A study conducted by Suleiman et al. revealed that the ethanolic extract of clove flower bud appeared to be rich in flavonoids (26.8%), phenolic acid (20.8%), and tannins (4.9%) [45], whose antioxidant effects were already well-known, similar to another phytochemical screening of clove made by Upadhyaya et al. [46]. In addition, the extract of clove flower bud with stronger antimicrobial capacity was also found to exhibit higher phenolic content [47], indicating that phenolic compounds that contributed to the antioxidant activity also displayed antibacterial capacity. Moreover, some components mainly existing in volatile oil also participated in the contribution of antibacterial activity, such as eugenol, isoeugenol, eugenyl acetate, caryophyllene, and humulene. Eugenol was even classified as a substance generally regarded as safe by Food and Drug Administration (FDA). Compared with male clove (flower bud), there were limited studies on female clove (fruit). Although they were derived from the same plant, chemical components were significantly different, and the phytochemicals in clove fruit were identified as eugenol, 2-hydroxy-4, 6-dimethoxy-5-methylacetophenone, and cyclohexane, which might exert antibacterial and antioxidant effects [48].

The antimicrobial activity of spice extracts is mainly attributed to their phytochemicals. Phenolic compounds, such as phenolic acids, flavonoids, and tannins are among the most abundant and widely distributed groups of secondary metabolites in edible plants [49,50]. Moreover, phenolic compounds have been reported to be highly responsible for the antioxidant activity in spices [9], which is also agreement with our results, showing strong correlation between TPC and FRAP/ABTS values (*r* = 0.918 and 0.931, respectively, *p* < 0.01). Thus, TPC can serve as a bridge connecting the antibacterial and antioxidant activity of spice extracts. In a previous study, Shan et al. showed that there was a strong positive linear relationship among antibacterial activity, antioxidant activity, and TPC values in spices [29]. Indeed, in some spices like sage, higher antibacterial activity could be observed in spices containing higher TPC [51]. Moreover, some phenolic compounds identified in spices showed good bacterial inhibitory efficiency. Taking oregano as an example, its antibacterial activity was strongly linked to the presence of phenolic compounds like carvacrol and thymol [52]. Besides, the phenolic compounds identified in many spices like curcumin in turmeric, eugenol in cloves, thymol in thyme, and gingerol in ginger, as well as caffeic acids and ferulic acids in thyme, cinnamon, and galangal have also been demonstrated to exhibit evident antibacterial capacity [8,50,53,54,55,56]. Moreover, the number and position of phenolic hydroxyl groups are also considered to be tightly related to the toxicity towards microorganisms [6]. The antibacterial activity of these phenolic compounds involves many modes of action, such as destroying cell membrane morphology, altering membrane fatty acids, depleting proton motive force, causing reactive oxygen damage, impairing enzymatic mechanisms for energy production and metabolism, disrupting normal functionality of proteins, and inhibiting nucleic acid synthesis [6,29,57].

In our study, however, we found a significant but weak correlation of antibacterial activity with TPC and antioxidant activity, indicating that polyphenols were only partially contributed to the antibacterial activity of spice extracts. The Pearson correlation coefficient (*r* = 0.541) tested between TPC and DIZ values of *S. aureus* in our study was overall consistent with a previous study [24], reporting that the TPC of 28 pigmented edible bean coats were weakly correlated (*r* = 0.540) with DIZ values of *S. aureus*. In addition, Weeakkody et al. found a similarly poor correlation (*r^2^* < 0.30) between the antimicrobial activity of seven edible spice extracts and phenolic compound levels [58]. Our study and these studies suggest that in addition to polyphenols, there should be other substances responsible for the overall antibacterial activity of spice extracts. For instance, in our study, although the TPC of galanga was lower than some other spices, its antibacterial activity was highest among tested spices, indicating that other nonphenolic constituents, like 5-hydroxymethyl furfural (accounting for 59.9% in methanol extract), might have the capacity to act as antimicrobial agents [59,60]. Besides, alkaloids, such as piperine from black pepper, were also found to be effective against *Escherichia coli*, *Klebsiella penumonia*, *Salmonella enterica*, and *S. aureus* [61]. Therefore, polyphenols combined with other bioactive compounds should contribute to the overall antibacterial activity of spice extracts.

## 5. Conclusions

This study investigated systematically the antibacterial activity, antioxidant activity, and TPC of 67 spice extracts. The antibacterial activity of the spice extracts was partially ascribed to polyphenols, while detailed contributions of other antibacterial components should be elucidated in future work. Five selected spice extracts showed the strongest antibacterial activity against different strains of antibiotic-resistant *S. aureus*, and they have potential for use as antibiotic alternatives in animal feeding. Moreover, the clove, exhibiting both excellent antioxidant and antibacterial activities, has great potential as a natural food preservative in the food industry.

## Figures and Tables

**Table 1:** Antibacterial properties of 67 spice extracts.

Scientific Name	Common Name	Parts Tested	Diameters of Inhibitory Zone (DIZ, mm)			
			*S. aureus* SJTUF 20978 (Resistant)	*S. aureus* ATCC 25923 (Normal)	*S. enteritidis* SJTUF 10987 (Resistant)	*S. enteritidis* ATCC 13076 (Normal)
*Alpinia galangal* (L.) Willd.	Galangal	Rhizome	25.6 ± 0.49	31.7 ± 0.21	NIZ	NIZ
*Alpinia galanga* Willd.	Fructus galangae	Fruit	20.2 ± 0.52	28.3 ± 0.29	NIZ	NIZ
*Alpinia hainanensis* K. Schum.	Semen alpiniae katsumadai	Fruit	14.8 ± 0.74	17.5 ± 0.09	NIZ	NIZ
*Alpinia officinarum* Hance	Small galangal	Rhizome	11.4 ± 0.24	11.9 ± 0.14	NIZ	NIZ
*Alpinia tonkinensis* Gagnep	Green gardamon	Fruit	NIZ	NIZ	NIZ	NIZ
*Amomum aurantiacum* H. T. Tsai et S. W. Zhao	Thorn amomum villosum	Fruit	13.4 ± 0.09	15.3 ± 0.24	8.6 ± 0.21	9.1 ± 0.24
*Amomum testaceum* Ridl	Fructus amomi rotundus	Fruit	12.0 ± 0.29	13.5 ± 0.29	NIZ	NIZ
*Amomum tsao-ko* Crevost et Lemarié	Fructus tsaoko	Fruit	14.1 ± 0.09	14.8 ± 0.24	NIZ	NIZ
*Amomum villosum* Lour.	Fructus amomi	Fruit	12.1 ± 0.24	14.9 ± 0.09	NIZ	NIZ
*Anethum graveolens* L.	Dill	Seed	NIZ	NIZ	NIZ	NIZ
*Angelica dahurica* (Hoffm.) Benth. et Hook.f. ex Franch. et Sav.	Radix angelicae formosanae	Rhizome	NIZ	NIZ	NIZ	NIZ
*Areca catechu* L.	Areca	Fruit	10.1 ± 0.09	9.90 ± 0.09	NIZ	NIZ
*Artemisia dracunculus* L.	Tarragon	Leaf	NIZ	NIZ	NIZ	NIZ
*Aucklandia lappa* Decne.	Costustoot	Rhizome	10.1 ± 0.19	15.2 ± 0.29	NIZ	NIZ
*Capsicum annuum* L.	Dry chilli (grown in Henan)	Fruit	NIZ	NIZ	NIZ	NIZ
*Capsicum annuum* L.	Dry chilli (grown in Sichuan)	Fruit	NIZ	NIZ	NIZ	NIZ
*Capsicum annuum* L.	Dry chilli (grown in Yunnan)	Fruit	NIZ	NIZ	NIZ	NIZ
*Capsicum annuum* var. grossum	Bell pepper	Fruit	NIZ	NIZ	NIZ	NIZ
*Carum carvi* L.	Caraway	Fruit	8.70 ± 0.09	10.6 ± 0.09	NIZ	NIZ
*Cinnamomum cassia* (L.) J.Presl	Cinnamon	Bark	20.7 ± 0.47	27.6 ± 1.73	16.0 ± 0.52	15.5 ± 0.38
*Citrus limon* (L.) Osbeck	Dried lemon	Fruit	11.3 ± 0.09	13.0 ± 0.09	NIZ	NIZ
*Citrus reticulata* Blanco	Citrus	Fruit	NIZ	NIZ	NIZ	NIZ
*Citrus reticulata* Blanco	Old citrus	Fruit	NIZ	NIZ	NIZ	NIZ
*Coriandrum sativum* L	Coriander	Fruit	NIZ	NIZ	NIZ	NIZ
*Crataegus pinnatifida* Bunge	Hawthorn	Fruit	11.4 ± 0.50	12.1 ± 0.29	NIZ	NIZ
*Cuminum cyminum* L.	Chinese cumin seed	Fruit	NIZ	NIZ	NIZ	NIZ
*Curcuma longa* L.	Turmeric	Rhizome	NIZ	NIZ	NIZ	NIZ
*Cymbopogon citratus* (DC.) Stapf.	Lemongrass	Leaf	NIZ	NIZ	NIZ	NIZ
*Eleutherococcus nodiflorus* (Dunn) S.Y.Hu.	Cortex acanthopanacis	Bark	NIZ	NIZ	NIZ	NIZ
*Foeniculum vulgare* Mill.	Fennel (traditional Chinese spice)	Fruit	NIZ	NIZ	NIZ	NIZ
*Foeniculum vulgare*	Kelly anise seeds (Western food spice)	Fruit	NIZ	NIZ	NIZ	NIZ
*Gardenia jasminoides* J. Ellis	Gardenia	Fruit	NIZ	NIZ	NIZ	NIZ
*Glycyrrhiza uralensis* Fisch.	Liquorice	Leaf	15.8 ± 0.14	15.6 ± 0.57	NIZ	NIZ
*Illicium verum* Hook. f.	Star anise	Fruit	12.5 ± 0.09	12.8 ± 0.29	NIZ	NIZ
*Kaempferia galanga* L.	Rhizoma kaempferiae	Rhizome	10.1 ± 0.49	10.3 ± 0.52	NIZ	NIZ
*Laurus nobilis* L.	Bay leaf	Leaf	12.5 ± 0.33	13.0 ± 0.09	NIZ	NIZ
*Lithospermum erythrorhizon* Sieb. et Zucc.	Lithospermum	Leaf	13.3 ± 0.21	13.3 ± 0.28	NIZ	9.6 ± 0.29
*Lysimachia capillipes* Hemsl	Nephrolepis	Stem	12.4 ± 0.45	13.9 ± 0.62	NIZ	NIZ
*Lysimachia foenum-graecum* Hance	Avandula pedunculata	Whole plant	12.3 ± 0.49	14.4 ± 0.33	NIZ	10.9 ± 0.24
*Magnolia denudata* Desr.	Magnolia flower	Flower	NIZ	NIZ	NIZ	NIZ
*Mentha canadensis* L.	Pepper mint	Leaf	12.5 ± 0.09	15.9 ± 0.33	NIZ	NIZ
*Monascus purpureus* Went	Red yeast rice	Fruit	NIZ	NIZ	NIZ	NIZ
*Murraya koenigii* (L.) Spreng.	Curry leaves	Leaf	9.80 ± 0.61	10.8 ± 0.09	NIZ	NIZ
*Murraya paniculata* (L.) Jack.	Murraya paniculata	Leaf	NIZ	NIZ	NIZ	NIZ
*Myristica fragrans* Houtt.	Semen myristicae	Fruit	10.5 ± 0.21	11.6 ± 0.29	NIZ	NIZ
*Nardostachys jatamansi* (D. Don) DC.	Nard	Stem	14.1 ± 0.47	15.5 ± 0.21	NIZ	NIZ
*Ocimum basilicum* L.	Basil	Leaf	NIZ	NIZ	NIZ	NIZ
*Origanum majorana* L.	Marjoram	Whole plant	17.2 ± 0.71	15.2 ± 0.62	NIZ	NIZ
*Origanum vulgare* L.	Origanum	Leaf	14.1 ± 0.39	11.8 ± 0.09	NIZ	NIZ
*Petroselinum crispum* (Mill.) Fuss	Parsley	Leaf	NIZ	NIZ	NIZ	NIZ
*Pimenta dioica* (L.) Merr.	Allspice	Fruit	13.2 ± 0.09	14.9 ± 0.19	NIZ	NIZ
*Piper longum* L.	Long pepper	Cluster	NIZ	NIZ	NIZ	NIZ
*Piper nigrum* L.	Black pepper	Fruit	NIZ	NIZ	NIZ	NIZ
*Piper nigrum* L.	White pepper	Fruit	NIZ	NIZ	NIZ	NIZ
*Piper nigrum* L.	Red pepper	Fruit	14.2 ± 0.14	16.8 ± 0.09	NIZ	NIZ
*Piper nigrum* L.	Green pepper	Fruit	NIZ	NIZ	NIZ	NIZ
*Reseda odorata* L.	Integrated vanilla	Leaf	14.7 ± 0.51	14.0 ± 0.75	NIZ	NIZ
*Rosmarinus officinalis* L.	Rosemary	Leaf	18.3 ± 0.21	20.8 ± 0.14	NIZ	10.3 ± 0.42
*Salvia japonica* Thunb.	Sage	Leaf	15.4 ± 0.49	19.3 ± 0.21	NIZ	NIZ
*Sinapis alba* L.	Yellow mustard seeds	Seed	18.6 ± 1.03	21.9 ± 0.16	NIZ	NIZ
*Sinapis alba* L.	Black mustard seeds	Seed	NIZ	NIZ	NIZ	NIZ
*Sophora alopecuroides* L.	Fenugreek	Fruit	NIZ	NIZ	NIZ	NIZ
*Syzygium aromaticum* (L.) Merr. et L. M. Perry	Male clove	Flower	15.6 ± 0.33	16.4 ± 0.39	11.0 ± 0.21	9.0 ± 0.29
*Syzygium aromaticum*	Female clove	Fruit	15.1 ± 0.49	20.9 ± 0.14	8.5 ± 0.24	10.6 ± 0.09
*Thymus vulgaris* L.	Thyme	Leaf	16.0 ± 0.33	14.9 ± 0.42	NIZ	NIZ
*Zanthoxylum bungeanum* Maxim	Red Chinese prickly ash	Fruit	11.3 ± 0.09	12.9 ± 0.21	NIZ	NIZ
*Zanthoxylum bungeanum* Maxim	Green Chinese prickly ash	Fruit	13.3 ± 0.24	13.3 ± 0.21	NIZ	NIZ

The data were averages of three measurements with standard deviation. NIZ means no inhibition zone.

**Table 2:** Verification of drug-resistant bacteria.

Name of Antibiotics	Class	Breakpoint Conc. (μg/mL)	*S. enteritidis* SJTUF 10987	*S. aureus* SJTUF										
				20745	20746	20755	20758	20772	20827	20841	20862	20973	20978	20991
Ampicillin	*β*-lactams	32	+											
Cefazolin		8												
Oxacillin		4												
Penicillin		0.25					+							
Gentamicin	Aminoglycosides	16			+			+		+	+	+		
Streptomycin		64	+	+		+	+						+	
Ciprofloxacin	Fluoroquinolones	4	+	+	+	+		+		+	+	+		+
Clindamycin	Lincosamides	4		+	+	+	+	+		+	+		+	+
Erythromycin	Macrolides	8		+	+	+	+	+	+	+	+		+	+
Sulfisoxazole	Sulfonamides	512	+											
Tetracycline	Tetracyclines	16												+

+ means bacterial growth.

**Table 3:** Minimum inhibitory concentration (MIC) and minimum bactericide concentration (MBC) values of selected 11 spice extracts with good antibacterial activity.

Scientific Name	Common Name	MIC (mg/mL)	MBC (mg/mL)
*Alpinia galangal* (L.) Willd.	Galangal	6.25	6.25
*Alpinia galanga* Willd.	Fructus galangae	6.25	6.25
*Cinnamomum cassia* (L.) J. Presl	Cinnamon	0.8	1.6
*Glycyrrhiza uralensis* Fisch.	Liquorice	0.4	0.8
*Origanum majorana* L.	Marjoram	1.6	1.6
*Rosmarinus officinalis* L.	Rosemary	0.4	0.4
*Salvia japonica* Thunb.	Sage	0.4	0.8
*Sinapis alba* L.	Yellow mustard seeds	6.25	12.5
*Syzygium aromaticum* (L.) Merr. et L. M. Perry	Male clove (flower)	0.8	1.6
*Syzygium aromaticum* (L.) Merr. et L. M. Perry	Female clove (fruit)	0.4	0.4
*Thymus vulgaris* L.	Thyme	1.6	1.6

**Table 4:** Antioxidant activity and total phenolic content of 67 spice extracts.

Scientific Name	Common Name	TPC (mg GAE/g DW)	FRAP (mmol Fe (II)/g DW)	TEAC (mmol Trolox/g DW)
*Alpinia galangal* (L.) Willd.	Galangal	119 ± 6.41	608 ± 55.6	394 ± 27.1
*Alpinia galanga* Willd.	Fructus galangae	122 ± 2.49	687 ± 30.8	423 ± 58.0
*Alpinia hainanensis* K. Schum.	Semen alpiniae katsumadai	473 ± 8.67	2876 ± 197	2662 ± 83.7
*Alpinia officinarum* Hance	Small galangal	281 ± 15.3	967 ± 43.5	707 ± 47.5
*Alpinia tonkinensis* Gagnep	Green gardamon	64.9 ± 4.72	617 ± 24.0	284 ± 19.5
*Amomum aurantiacum* H. T. Tsai et S. W. Zhao	Thorn amomum villosum	350 ± 11.3	3433 ± 137	1836 ± 127
*Amomum testaceum* Ridl	Fructus amomi rotundus	84.7 ± 2.67	1220 ± 99.6	288 ± 25.4
*Amomum tsao-ko* Crevost et Lemarié	Fructus tsaoko	303 ± 0.78	2542 ± 184	1902 ± 123
*Amomum villosum* Lour.	Fructus amomi	360 ± 6.80	3605 ± 201	2153 ± 370
*Anethum graveolens* L.	Dill	115 ± 1.83	867 ± 51.2	234 ± 11.3
*Angelica dahurica* (Hoffm.) Benth. et Hook.f. ex Franch. et Sav.	Radix angelicae formosanae	16.6 ± 0.48	208 ± 20.9	75.3 ± 8.74
*Areca catechu* L.	Areca seed	95.9 ± 3.71	741 ± 35.8	390 ± 41.9
*Artemisia dracunculus* L.	Tarragon leaf	148 ± 5.40	1118 ± 3.15	461 ± 33.7
*Aucklandia lappa* Decne.	Costustoot	21.7 ± 1.90	288 ± 7.51	105 ± 3.65
*Capsicum annuum* L.	Dry chilli (grown in Henan)	28.4 ± 1.55	160 ± 7.16	99.2 ± 16.8
*Capsicum annuum* L.	Dry chilli (grown in Sichuan)	17.3 ± 0.14	142 ± 4.26	59.6 ± 5.20
*Capsicum annuum* L.	Dry chilli (grown in Yunnan)	29.2 ± 1.59	270 ± 5.45	109 ± 4.98
*Capsicum annuum* var. grossum	Bell pepper	16.1 ± 3.18	105 ± 12.7	77.2 ± 7.52
*Carum carvi* L.	Caraway	42.3 ± 1.62	369 ± 33.3	165 ± 54.0
*Cinnamomum cassia* (L.) J.Presl	Cinnamon	349 ± 12.0	3013 ± 99.0	1857 ± 47.9
*Citrus limon* (L.) Osbeck	Dried lemon	7.35 ± 0.68	50.3 ± 2.32	17.4 ± 0.62
*Citrus reticulata* Blanco	Citrus	39.7 ± 1.10	233 ± 11.4	241 ± 9.14
*Citrus reticulata* Blanco	Old citrus	68.2 ± 2.40	306 ± 13.8	218 ± 12.3
*Coriandrum sativum* L	Coriander	31.4 ± 0.42	306 ± 2.84	111 ± 9.41
*Crataegus pinnatifida* Bunge	Hawthorn	98.9 ± 2.09	768 ± 46.4	414 ± 10.1
*Cuminum cyminum* L.	Chinese cumin seed	58.8 ± 2.08	465 ± 18.6	184 ± 8.84
*Curcuma longa* L.	Turmeric	251 ± 4.30	1444 ± 51.5	1489 ± 252
*Cymbopogon citratus* (DC.) Stapf.	Lemongrass	153 ± 1.71	1586 ± 96.9	510 ± 34.5
*Eleutherococcus nodiflorus* (Dunn) S.Y.Hu.	Cortex acanthopanacis	79.1 ± 3.64	704 ± 55.1	372 ± 5.41
*Foeniculum vulgare* Mill.	Fennel (traditional Chinese spice)	58.1 ± 2.36	365 ± 16.4	222 ± 27.9
*Foeniculum vulgare*	Kelly anise seeds (Western spice)	30.5 ± 2.99	356 ± 8.33	221 ± 36.9
*Gardenia jasminoides* J. Ellis	Gardenia	45.4 ± 3.92	553 ± 17.4	152 ± 8.22
*Glycyrrhiza uralensis* Fisch.	Liquorice	65.8 ± 3.08	363 ± 13.0	365 ± 23.7
*Illicium verum* Hook. f.	Star anise	165 ± 5.00	1650 ± 35.5	855 ± 43.0
*Kaempferia galanga* L.	Rhizoma kaempferiae	15.9 ± 0.14	65.3 ± 2.73	23.0 ± 0.67
*Laurus nobilis* L.	Bay leaf	182 ± 1.46	1255 ± 81.9	1124 ± 100
*Lithospermum erythrorhizon* Sieb. et Zucc.	Lithospermum	80.9 ± 5.89	627 ± 6.44	325 ± 12.9
*Lysimachia capillipes* Hemsl	Nephrolepis	79.1 ± 0.91	709 ± 39.4	306 ± 32.2
*Lysimachia foenum-graecum* Hance	Avandula pedunculata	98.5 ± 8.57	759 ± 45.7	366 ± 32.8
*Magnolia denudata* Desr.	Magnolia flower	63.2 ± 3.71	612 ± 18.6	244 ± 10.1
*Mentha canadensis* L.	Pepper mint	280 ± 2.97	3180 ± 167	1296 ± 29.5
*Monascus purpureus* Went	Red yeast rice	65.8 ± 2.32	224 ± 5.94	158 ± 36.7
*Murraya koenigii* (L.) Spreng.	Curry leaves	146 ± 5.43	463 ± 27.9	241 ± 8.47
*Murraya paniculata* (L.) Jack.	Murraya paniculata	70.4 ± 1.09	497 ± 1.36	349 ± 86.9
*Myristica fragrans* Houtt.	Semen myristicae	111 ± 3.66	934 ± 27.3	552 ± 21.7
*Nardostachys jatamansi* (D. Don) DC.	Nard	103 ± 2.08	553 ± 29.4	219 ± 29.0
*Ocimum basilicum* L.	Basil	147 ± 4.71	1410 ± 24.6	487 ± 12.3
*Origanum majorana* L.	Marjoram	303 ± 3.92	3272 ± 130	1563 ± 125
*Origanum vulgare* L.	Origanum	204 ± 0.69	2164 ± 135	714 ± 21.0
*Petroselinum crispum* (Mill.) Fuss	Parsley	58.4 ± 1.89	521 ± 24.1	245 ± 34.8
*Pimenta dioica* (L.) Merr.	Allspice	339 ± 1.36	4404 ± 23.9	2184 ± 43.9
*Piper longum* L.	Long pepper	92.0 ± 1.20	1733 ± 68.9	789 ± 44.2
*Piper nigrum* L.	Black pepper	64.3 ± 7.74	576 ± 13.0	239 ± 9.82
*Piper nigrum* L.	White pepper	36.8 ± 0.96	354 ± 9.54	251 ± 50.7
*Piper nigrum* L.	Red pepper	378 ± 3.52	4137 ± 147	2055 ± 76.4
*Piper nigrum* L.	Green pepper	73.5 ± 3.91	699 ± 33.2	262 ± 9.39
*Reseda odorata* L.	Integrated vanilla	269 ± 5.74	2671 ± 120	801 ± 42.9
*Rosmarinus officinalis* L.	Rosemary	261 ± 9.29	2712 ± 160	821 ± 20.8
*Salvia japonica* Thunb.	Sage	204 ± 3.17	2345 ± 186	700 ± 14.2
*Sinapis alba* L.	Yellow mustard seeds	183 ± 2.77	599 ± 29.6	240 ± 4.22
*Sinapis alba* L.	Black mustard seeds	53.2 ± 1.97	523 ± 15.0	229 ± 14.8
*Sophora alopecuroides* L.	Fenugreek	39.8 ± 2.33	235 ± 23.8	104 ± 9.41
*Syzygium aromaticum* (L.) Merr. et L. M. Perry	Male clove (flower)	424 ± 14.9	5453 ± 23.9	3131 ± 177
*Syzygium aromaticum* (L.) Merr. et L. M. Perry	Female clove (fruit)	485 ± 18.5	6682 ± 68.6	3415 ± 53.1
*Thymus vulgaris* L.	Thyme	241 ± 14.7	3244 ± 143	743 ± 20.0
*Zanthoxylum bungeanum* Maxim	Red Chinese prickly ash	168 ± 6.58	1844 ± 29.7	1444 ± 245
*Zanthoxylum bungeanum* Maxim	Green Chinese prickly ash	193 ± 12.8	1961 ± 35.4	1398 ± 82.6

The data were averages of three measurements with standard deviation. TPC, total phenolic content; FRAP, ferric-reducing antioxidant power; TEAC, trolox equivalent antioxidant capacity.

**Table 5:** Correlation analysis among antibacterial activity, antioxidant activity, and total phenolic content.

Pearson Correlation Coefficient (*r*)	DIZ Value (*S. aureus* ATCC 25923)	DIZ Value (*S. aureus* SJTUF 20978)	TPC	FRAP	TEAC
DIZ value (*S. aureus* ATCC 25923)	1	0.956 (*p* < 0.001)	0.541 (*p* < 0.001)	0.466 (*p* < 0.001)	0.448 (*p* < 0.001)
DIZ value (*S. aureus* SJTUF 20978)		1	0.568 (*p* < 0.001)	0.490 (*p* < 0.001)	0.448 (*p* < 0.001)
TPC			1	0.919 (*p* < 0.001)	0.931 (*p* < 0.001)
FRAP				1	0.924 (*p* < 0.001)

Pearson correlation analysis was performed to analyze the relationships among the means of parameters. *p* < 0.01 was defined as statistical significance.

## Supplementary Materials

The following are available online at https://www.mdpi.com/2076-2607/7/6/157/s1: Table S1: The calculated general score of 67 extracts of spices.
