# Supplementary material for: Discovery of Antibacterial Dietary Spices That Target Antibiotic-Resistant Bacteria
Source: Microorganisms. 2019 May 29;7(6):157. doi: 10.3390/microorganisms7060157 (PMC6617121; doi:10.3390/microorganisms7060157)
Supplement: Supplementary file 1 [file microorganisms-07-00157-s001.pdf]

Table S1. The calculated general score of 67 spice extracts.

Sample (common name)	Results of PCA			
	C1	C2	GS	Ranking
Female clove	3.49	0.364	2.17	1
Male clove	2.94	0.126	1.75	2
Cinnamon	0.848	2.49	1.54	3
Senmen alpiniae katsumadai	2.11	0.421	1.40	4
Red pepper	1.92	0.305	1.24	5
Allspice	2.04	-0.054	1.15	6
Fructus amomi	1.90	-0.153	1.03	7
Thorn amomum villosum	1.60	0.147	0.987	8
Marjoram	1.14	0.743	0.971	9
Rosemary	0.258	1.70	0.867	10
Galangal	-1.56	4.16	0.860	11
Fructus tsaoko	1.24	0.273	0.831	12
Pepper mint	1.06	0.244	0.712	13
Thyme	0.575	0.730	0.640	14
Fructus galangae	-1.21	3.05	0.590	15
Integrated vanilla	0.563	0.621	0.587	16
Sage	0.083	1.21	0.559	17
Green Chinese prickly ash	0.572	0.188	0.410	18
Yellow mustard seed	-0.903	2.16	0.393	19
Origanum	0.314	0.251	0.287	20
Red Chinese prickly ash	0.579	-0.129	0.280	21
Bay leaf	0.236	0.160	0.204	22
Star anise	0.164	0.159	0.162	23
Small galangal	0.298	-0.051	0.151	24
Turmeric	1.01	-1.15	0.096	25
Liquorice	-0.946	1.16	-0.058	26
Nard	-0.795	0.905	-0.076	27
Avandula pedunculata	-0.575	0.503	-0.120	28
Fructus amomi rotundus	-0.486	0.340	-0.137	29
Lithospermum	-0.688	0.549	-0.165	30
Nephrolepis	-0.662	0.474	-0.181	31
Semen myristicae	-0.239	-0.110	-0.184	32
Hawthorn	-0.440	0.114	-0.206	33
Lemon grass	0.256	-0.945	-0.252	34
Long pepper	0.269	-0.985	-0.261	35
Basil	0.180	-0.924	-0.287	36
Curry leaves	-0.381	-0.212	-0.310	37

Areca seed	-0.355	-0.289	-0.327	38
Tarragon leaf	0.087	-0.895	-0.328	39
Costustoot	-1.00	0.382	-0.417	40
Dill	-0.199	-0.819	-0.461	41
Dried lemon	-1.14	0.368	-0.500	42
Cortex acanthopanacis	-0.274	-0.817	-0.503	43
Green pepper	-0.349	-0.794	-0.537	44
Caraway	-0.698	-0.324	-0.540	45
Murraya paniculata	-0.369	-0.792	-0.548	46
Green cardamon	-0.386	-0.787	-0.556	47
Magnolia flower	-0.413	-0.779	-0.568	48
Black pepper	-0.423	-0.775	-0.572	49
Parsley	-0.452	-0.770	-0.586	50
Black mustard seed	-0.474	-0.765	-0.597	51
Rhizoma kaempferiae	-0.979	-0.089	-0.603	52
Old citrus	-0.498	-0.751	-0.605	53
Chinese cumin seed	-0.498	-0.754	-0.606	54
Fennel	-0.508	-0.752	-0.611	55
Gardenia	-0.529	-0.750	-0.622	56
Red yeast rice	-0.559	-0.732	-0.632	57
White pepper	-0.557	-0.749	-0.638	58
Citrus	-0.588	-0.738	-0.651	59
Kelly anise seeds	-0.590	-0.741	-0.654	60
Fenugreek	-0.659	-0.712	-0.681	61
Coriander	-0.659	-0.717	-0.683	62
Yunnan dry chilli	-0.677	-0.712	-0.692	63
Henan dry chilli	-0.715	-0.701	-0.709	64
Radix angelicae formosanae	-0.748	-0.696	-0.726	65
Erjingtiao chilli	-0.772	-0.688	-0.737	66
Bell chilli	-0.777	-0.687	-0.739	67
